# Novel mGluR- and CB1R-Independent Suppression of GABA Release Caused by a Contaminant of the Group I Metabotropic Glutamate Receptor Agonist, DHPG

**DOI:** 10.1371/journal.pone.0006122

**Published:** 2009-07-01

**Authors:** Carlos A. Lafourcade, Longhua Zhang, Bradley E. Alger

**Affiliations:** 1 Department of Physiology, Program in Neuroscience, University of Maryland School of Medicine, Baltimore, Maryland, United States of America; 2 Facultad de Medicina, Universidad de Buenos Aires, Buenos Aires, Argentina; INSERM U862, France

## Abstract

**Background:**

Metabotropic glutamate receptors (mGluRs) are ubiquitous throughout the body, especially in brain, where they mediate numerous effects. MGluRs are classified into groups of which group I, comprising mGluRs 1 and 5, is especially important in neuronal communication. Group I actions are often investigated with the selective agonist, S-3,5-dihydroxyphenylglycine (DHPG). Despite the selectivity of DHPG, its use has often led to contradictory findings. We now report that a particular commercial preparation of DHPG can produce mGluR-independent effects. These findings may help reconcile some discrepant reports.

**Methods:**

We carried out electrophysiological recordings in the rat *in vitro* hippocampal slice preparation, focusing mainly on pharmacologically isolated GABA_A_-receptor-mediated synaptic currents. Principal Findings: While preparations of DHPG from three companies suppressed GABAergic transmission in an mGluR-dependent way, one batch had an additional, unusual effect. Even in the presence of antagonists of mGluRs, it caused a reversible, profound suppression of inhibitory transmission. This mGluR - independent action was not due to a higher potency of the compound, or its ability to cause endocannabinoid-dependent responses. Field potential recordings revealed that glutamatergic transmission was not affected, and quantal analysis of GABA transmission confirmed the unusual effect was on GABA release, and not GABA_A_ receptors. We have not identified the responsible factor in the DHPG preparation, but the samples were 99% pure as determined by HPLC and NMR analyses.

**Conclusions:**

In certain respects our observations with the anomalous batch strikingly resemble some published reports of unusual DHPG effects. The present findings could therefore contribute to explaining discrepancies in the literature. DHPG is widely employed to study mGluRs in different systems, hence rigorous controls should be performed before conclusions based on its use are drawn.

## Introduction

The synthetic amino acid S-3,5-dihydroxyphenylglycine (DHPG) is a potent group-I-selective mGluR agonist [1]that is widely used in areas of research as diverse as pain [2] cancer [3], drug abuse [4] and learning [5]. Activation of group I mGluRs by DHPG affects synaptic transmission in various ways [6], including the mobilization of endogenous cannabinoids (endocannabinoids, eCBs [7], [8]) and induction of eCB – mediated forms of short and long term synaptic plasticity [9], [10] by activating the cannabinoid receptor, CB1R. Despite its extensive use, DHPG sometimes produces controversial results, leading to variation in its reported potency and the degree to which antagonists of mGluRs and CB1Rs can oppose its functional actions, e.g., [11]–[16]. We have tested the hypothesis that some commercial preparations of DHPG harbor a chemical activity that can cause mGluR-independent actions. We compared the actions of DHPG from three different companies (Ascent Scientific, Sigma-Aldrich and Tocris Bioscience) on well-established bioassays of mGluR-mediated effects in the *in vitro* hippocampal slice. Multiple samples from one batch of DHPG obtained from Ascent Scientific transiently suppressed hippocampal GABAergic transmission in an mGluR- and CB1R-independent manner, whereas another batch from this source and batches from the other sources did not. We have not fully identified the contaminant responsible for the anomalous effects. It could not be distinguished from DHPG by HPLC, and may have a distinctive signature by proton NMR. The unrecognized presence of such effects could explain some controversial findings regarding mGluR control of synaptic transmission that have been reported. Finally, the ability of the unknown factor to reduce GABA, but not glutamate, release suggests that its identification may be of scientific interest in its own right.

## Results

### Comparison of the maximal potency of different batches of DHPG

We began by comparing the abilities of (S)-3,5 DHPG from three commercial sources – Ascent Scientific, Tocris, and Sigma-Aldrich – to suppress inhibitory synaptic transmission to pyramidal cells in CA1 region of the hippocampal slice. For convenience the drugs are designated A-DHPG, T-DHPG, and S-DHPG in the figures. Furthermore, we distinguish between batches Asc-08007-1-1 and Asc-08116-5-3 from Ascent Scientific; Asc-08007-1-1 was used throughout the study, except as noted.

Evoked inhibitory postsynaptic currents (eIPSCs) were produced in CA1 pyramidal cells by stimulating in CA3 in the presence of 2,3-Dioxo-6-nitro-1,2,3,4-tetrahydrobenzo[f]quinoxaline-7-sulfonamide (NBQX, 10 µM) and D-(-)-2-Amino-5-phosphonopentanoic acid (D-AP5, 20 µM), using either KGluconate (KGluc) - or KCl-based electrode solutions (Materials and Methods). Responses were evoked continuously at 0.25 Hz throughout the experiments. The outward eIPSCs recorded with the KGluc electrodes were smaller than the inward eIPSCs because of the smaller driving force, but otherwise the recording conditions were the same.

**Figure 1:**
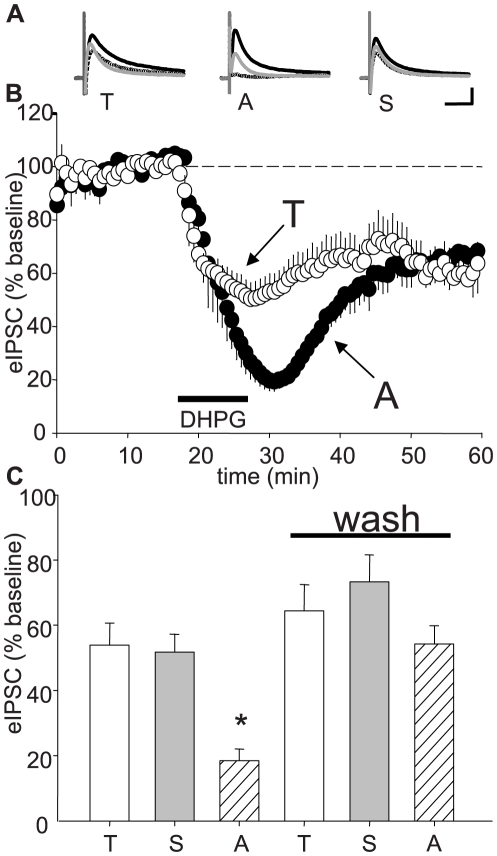
Comparison of eIPSC suppression caused by DHPG from different sources. (A) Representative traces showing how eIPSCs recorded with KGluc-filled electrodes were affected by 10-min applications of DHPG from Tocris Bioscience (T, left), Ascent Scientific, Asc-08007-1-1 (A, middle), or Sigma Aldrich (S, right). In this and all other experiments except those in Fig. 4, S-3,5 DHPG was applied at 50 µM. Black trace  =  baseline, dashed trace  =  DHPG, gray trace  = 25 min washout. Each trace is the average of ten consecutive responses. Cal. bars: y:100 pA, x: 50 ms. Results are expressed as percent of baseline eIPSC amplitudes. (B) Group data obtained with T-DHPG (white circles) or Asc-08007-1-1 (black circles) DHPG; S-DHPG data were omitted from the graph for clarity. (C) Summary of peak eIPSC depressions: T-DHPG: 54.0±6.7%, n = 8, p<0.01; S-DHPG: 51.7±5.5, n = 8, p<0.01, and Asc-08007-1-1, 18.5±3.6%, n = 5, p<0.001). Late eIPSC depressions (iLTD) were measured after 25-min DHPG washout (Wash) period. T-DHPG: 64.4±8.0%, n = 7, p<0.01; S-DHPG: 73.4±8.2, n = 5, p<0.05, and Asc-08007-1-1, 54.2±5.6%, n = 5, p<0.05). The effect of Asc-08007-1-1 differed significantly from the others during DHPG application (asterisk, p<0.05) but not after a 25-min washout.

DHPG was bath-applied at a maximal concentration of 50 µM for 10 min. All samples of DHPG triggered an initial strong depression of synaptic activity that recovered only partially after washout and remained at a reduced level for the duration of the recordings (≥25 min). The peak eIPSC decreases expressed as percent of baseline eIPSC amplitude occurred during or slightly after agonist application. Peak decreases were to ∼50% of baseline for T-DHPG and S-DHPG, but were significantly larger (p<0.05), to ∼20% of baseline for Asc-08007-1-1 (Figs. 1B, 1C). The persistent suppression, called inhibitory long-term depression (iLTD), was measured at 25 min of washout of DHPG and had the same properties as previously reported [10]. There were no significant differences in iLTD magnitude caused by the various DHPG batches (Fig. 1C).

### Effects of mGluR antagonists on different batches of DHPG

**Figure 2 pone-0006122-g002:**
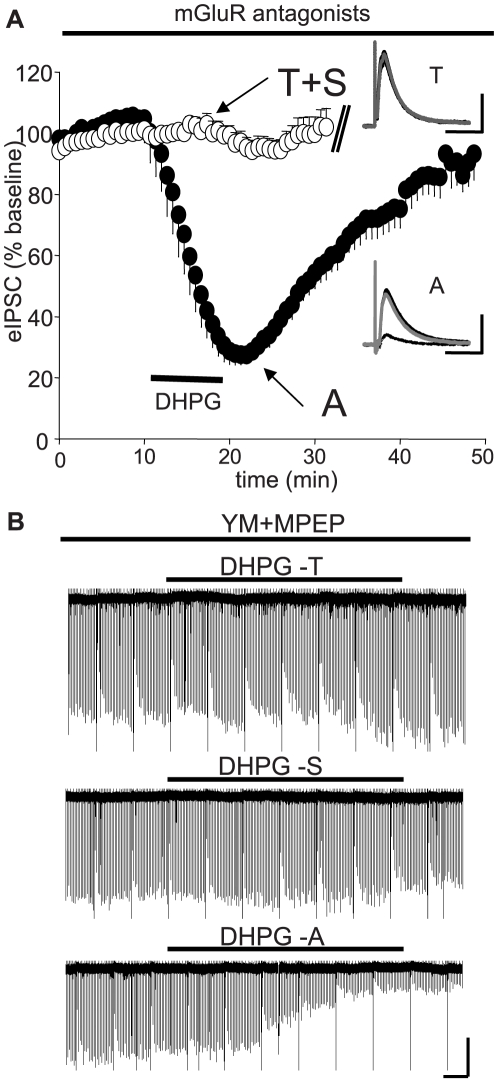
Inhibition of DHPG-induced iLTD by group I mGluR antagonists. The selective mGluR1 antagonist, YM298198 (4 µM) and the mGluR5 antagonist, MPEP (10 µM), were present throughout all experiments. (A) For display and statistical comparison among groups, the data from T-DHPG and S-DHPG were pooled (white circles). As no significant depression was observed with these compounds, recordings were stopped after 10 min of washout. Peak eIPSC depressions as percent of baseline for each drug individually (data not shown): T-DHPG: 95.4±6.3%, n = 5, n.s.; S-DHPG: 93.9±1.8%, n = 3, n.s.; Asc-08007-1-1: 30.7±5.2%, n = 7, p<0.001. Late eIPSC depressions (after 10 min washout): T-DHPG: 100.3±9.8%, n = 4, n.s.; S-DHPG: 101.8±5.6%, n = 3, n.s.; Asc-08007-1-1: 40.6±4.1%, n = 7, p<0.001. After 25-min of washout, there was no significant depression caused by Asc-08007-1-1 DHPG (black circles): 85.5±4.8%, n = 7, n.s. Insets: Representative traces: black trace  =  baseline, dashed trace  =  DHPG, gray trace  = 25 min washout. Each trace is the average of ten consecutive responses; KGluc-based electrode solution was used (Materials and Methods). Cal. bars: y:100 pA, x: 50 ms. (B) Continuous recorder trace showing the effects of T-DHPG, S-DHPG, and Asc-08007-1-1 DHPG sequentially applied, after washout of the previous appolication, to the same cell. A KCl-based electrode solution was used, so the eIPSCs are downward deflections that appear as straight lines at this time resolution. One-s voltage steps were given every 90 s to elicit Ca^2+^ influx through voltage-gated Ca^2+^ channels (VGCCs) and DSI [13], which appears as the transient reductions of eIPSCs. For display purposes, a small portion of the trace is omitted after each drug's washout. Note the strong remaining effect of Asc-08007-1-1, compared to the lack of effect of T-DHPG and S-DHPG. Cal. bars: y: 200 pA, x: 1 min.

The marked initial depression caused by the Asc-08007-1-1 might be explained by a greater potency of this drug for group I mGluRs, or perhaps another effect unrelated to mGluRs. To distinguish among these possibilities, we used potent group I mGluR antagonists, which have been repeatedly found to block all actions of DHPG in a variety of settings [13], [17]–[20]. The selective group I mGluR antagonists, 6-Amino-N-cyclohexyl-3-methylthiazolo[3,2-a]benzimidazole-2-carboxamide (YM298198, for mGluR1) and 2-Methyl-6-(phenylethynyl)pyridine hydrochloride (MPEP, for mGluR5), completely blocked the effects of T- and S-DHPG, as no significant eIPSC suppression was observed during application or washout of DHPG when they were present. Since the values for S- and T-DHPG do not differ significantly, they were pooled in Fig. 2A. In contrast, even in the presence of these group I mGluR antagonists, Asc-08007-1-1 caused a very substantial depression in peak eIPSC amplitudes, to ∼30% of baseline level. The depression was still present after a 10-min washout, but not after a 25-min washout (Fig. 2A). Figure 2B illustrates that the differences between Asc-08007-1-1 and the T- and S-DHPG can be demonstrated within the same cell. In this experiment (n = 5) all three DHPGs were applied sequentially in the presence of mGluR antagonists. Only Asc-08007-1-1 suppressed eIPSCs in these conditions. Taken together, the results suggest that Asc-08007-1-1 has an early, group I mGluR-independent, suppressive effect on eIPSCs. We considered the possibility that the residual effect of Asc-08007-1-1 was mediated by another type of mGluR, and so we also compared its effects in the presence of the non-selective mGluR blocker (2S)-2-Amino-2-[(1S,2S)-2-carboxycyclorop-1-yl]-3-(xan th-9-yl) propanoic acid (LY341495) [18]. LY341495 also failed to inhibit the eIPSC suppression caused by Asc-08007-1-1, implying that the early transient effect is independent of all mGluRs.

### CB1R antagonists do not block actions of Asc-08007-1-1

**Figure 3:**
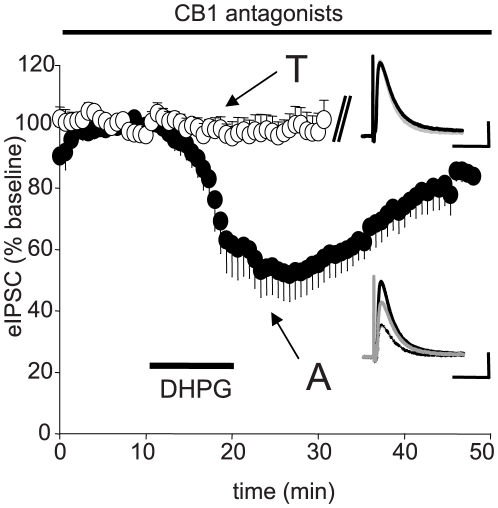
Inhibition of iLTD by CB1R antagonists. AM251 or SR141716A was present throughout all experiments. T-DHPG (T, white circles), Asc-08007-1-1, A, black circles). As T-DHPG caused no significant depression, recordings were stopped after 10 min of washout. Insets show representative traces for each condition. Black trace  =  baseline, dashed trace  =  DHPG, gray trace  = 25 min washout. Each trace is the average of ten consecutive responses. Peak eIPSC depression expressed as percent of baseline: T-DHPG 98.3±5.3%, n = 7, n.s.; Asc-08007-1-1: 53.6±8.7%, n = 6, p<0.01. Late eIPSC depressions (10-min washout); T-DHPG: 99.8±8.3%, n = 6, n.s.; Asc-08007-1-1: 57.5±7.6%, n = 6, p<0.05. Peak eIPSC was not significant from baseline after a 25 min washout of Asc-08007-1-1: 85.8±2.1%, n = 4, n.s. Cal. bars: y:100 pA, x: 50 ms.

In view of contradictory reports on the ability of mGluR antagonists to prevent CB1R-dependent actions of mGluRs (cf [11], [12]), it was important to test the CB1R-dependent effects of DHPG from another source against those of Asc-08007-1-1. If Asc-08007-1-1 does mediate transient, mGluR-independent effects, then it should also mediate transient, CB1R-independent effects. In confirmation of previous reports, we found that N-(Piperidin-1-yl)-5-(4-iodophenyl)-1-(2,4-dichlorophen yl)-4-methyl-1H-pyrazole-3-carboxamide (AM251, 4 µM) or 5-(4-Chlorophenyl)-1-(2,4-dichloro-phenyl)-4-methyl-N-(piperdin-1-yl)-1H-pyrazole-3-carboxamide (SR 141716A, 2 µM) completely blocked the acute effects of T-DHPG on eIPSCs (Fig. 3). Neither short- nor long-term eIPSC depression occurred. However, in the presence of a CB1R antagonist, Asc-08007-1-1 caused a significant transient depression to ∼50% of the baseline eIPSC. Again, no significant depression remained after 25 min of washout (Fig. 3).

### Unspecific effects of Asc-08007-1-1 are seen at low concentrations

**Figure 4:**
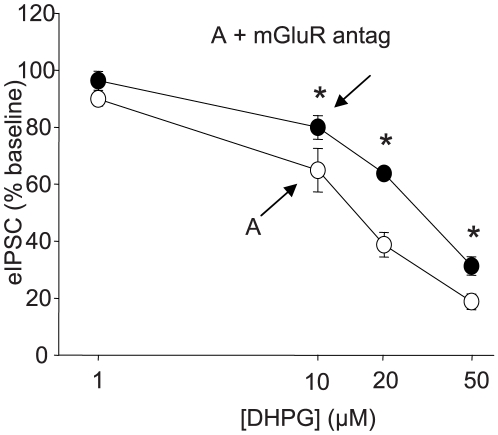
Concentration-response curves for Asc-08007-1-1 DHPG. Concentration-response curves in the presence (black circles) or absence (white circles) of mGluR antagonists (YM298198 plus MPEP, or LY341495 alone). Peak eIPSC amplitude depressions expressed as percent of baseline. Depression caused by all DHPG concentrations >1 µM are significant (p<0.01) in the presence and absence of antagonists. White circles: 1 µM: 89.9±2.5%, n = 4; 10 µM: 64.9±7.6%, n = 6; 20 µM: 38.8±4.3%, n = 6; 50 µM: 18.8±2.7%, n = 4. Black circles: 1 µM 96.4±3.3%, n = 4; 10 µM: 80.0±4.2%, n = 5; 20 µM: 63.8±1.3%, n = 6; 50 µM: 31.3±3.3%, n = 6. The difference between the depressions observed in the presence or absence of antagonists is significant for concentrations >10 µM (p<0.05).

We considered that the unexpected effects of Asc-08007-1-1 could be related to the concentration used. Our standard dose, 50 µM, was chosen to be functionally maximal and it seemed possible that lower concentrations of Asc-08007-1-1 would have effects that would be fully blocked by mGluR antagonists. To test this, we compared the effects of Asc-08007-1-1 at concentrations of 1, 10, 20, and 50 µM, each applied for 5 min, in the presence or absence of YM298198 plus MPEP, or LY341495 alone (Fig. 4). At all concentrations >1 µM, Asc-08007-1-1 significantly reduced eIPSC amplitudes either in the presence or absence of mGluR antagonists. The antagonists did decrease Asc-08007-1-1 effects, as concentrations >10 µM were less efficacious (p<0.05) in their presence than in control solution. Probably the greater variability of the 10 µM dose in control solution accounts for the lack of statistically significant antagonism at this concentration. In any case, the results show that Asc-08007-1-1 has mGluR-antagonist-resistant effects even at low concentrations.

### Only inhibitory synaptic transmission is sensitive to the unusual action of ASC-08007-1-1

**Figure 5:**
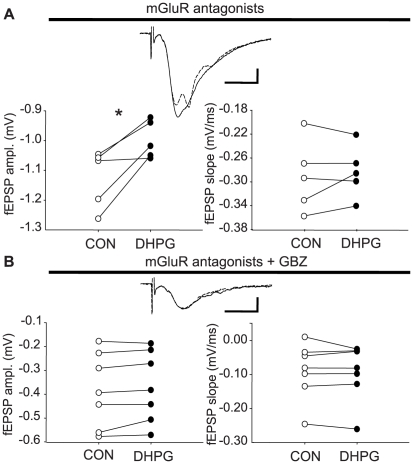
Asc-08007-1-1 DHPG does not affect fEPSPs in the presence of mGluR antagonists and gabazine. Traces in (A) and (B) show representative fEPSPs for each condition; NBQX and AP5 absent, and YM298198 and MPEP present for all experiments. Each trace (black  =  baseline, dashed  =  DHPG) is the average of 10 responses. (A) Left graph: raw amplitude measurement of fEPSPs before (control, n = 5) or during Asc-08007-1-1 application (DHPG, n = 5). Right graph: raw slope measurement of fEPSPs before (control, n = 5) or during DHPG application (DHPG, n = 5). (B) Same as in (A), but fEPSPs were recorded in the presence of gabazine, 20 µM. Left graph: raw amplitude measurement of fEPSPs before (control, n = 7) or during Asc-08007-1-1 application (DHPG, n = 7). Right graph: raw slope measurement of fEPSPs before (control, n = 7) or during Asc-08007-1-1 application (DHPG, n = 7, n.s.). Cal. bars: y: 0.1mV, x: 10 ms. Asterisk: significant difference, p<0.05, paired t-test.

Thus far the data reveal an mGluR- and CB1R-independent suppression of eIPSCs by Asc-08007-1-1. The question arises as to the nature of the cellular mechanism by which Asc-08007-1-1 affects synaptic transmission. If it is a general, non-specific block of a fundamental step in the transmitter release process, then Asc-08007-1-1 should reduce excitatory synaptic transmission as well. To test this prediction, we recorded excitatory fEPSPs that are mediated largely by AMPARs in CA1 *s. radiatum*. NBQX and AP5 were absent and mGluR antagonists YM298198 and MPEP were present in these experiments. After a 5-min application of Asc-08007-1-1 (50 µM) the field potentials showed evidence of hyperexcitability in the form of population spike potential oscillations on the wave. The fEPSP amplitudes appeared to be decreased (to 88.9±0.9% of baseline, p<0.05, n = 5; Fig. 5A), but this is probably attributable to interference from the population spikes. The slope of the fEPSP (measured at 1–1.5 ms after the fiber volley) on the other hand, did not change during Asc-08007-1-1 application (98.5±1.5% of baseline, n = 5). The apparent lack of effect on the excitatory synapses themselves, together with the increase in excitability seen in the repetitive population spike discharge could be explained by a selective effect on inhibitory transmission. The hyperexcitable state made it difficult to examine excitatory synaptic responses directly, however. We therefore recorded field potentials in the presence of the GABA_A_ channel blocker, 6-Imino-3-(4-methoxyphenyl)-1(6H)-pyridazinebutanoic acid hydrobromide (gabazine, 20 µM). For these experiments, the CA3 region was removed from the slice and in some cases small amounts of ionotropic GluR antagonists were used to reduce hyperexcitability (Materials and Methods). If Asc-08007-1-1 increases excitability only by suppressing inhibition, then it should have no effect when GABAergic synapses are blocked. We found that, indeed, in the presence of gabazine and group I mGluR antagonists Asc-08007-1-1 had no significant influence on the amplitudes (97±0.9% of baseline, n = 7) or the slope (94.7±2.7% of baseline, n = 7; Fig. 5B). It appears that the mGluR- and CB1R- independent effects of Asc-08007-1-1 only affect GABAergic synapses.

### The unexpected effects of Asc-08007-1-1 target GABA release

**Figure 6:**
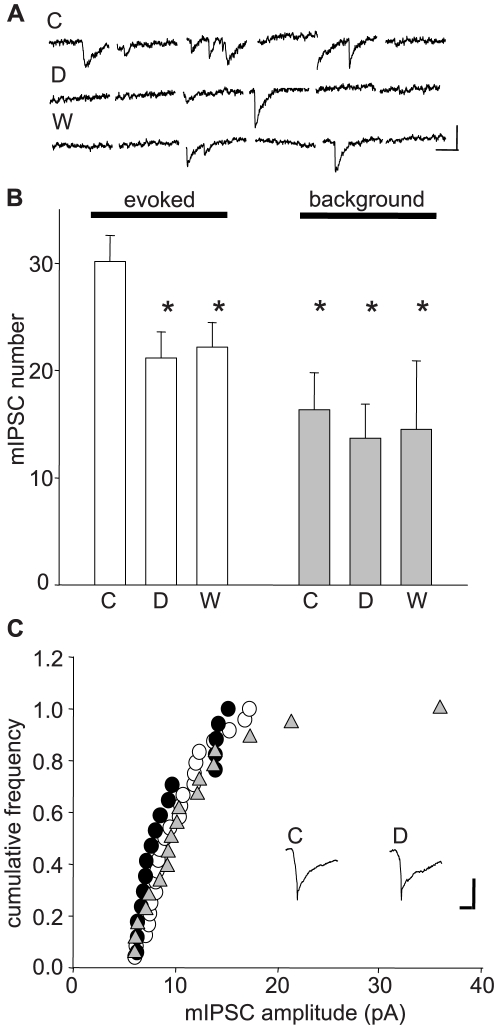
Asc-08007-1-1 DHPG reduces frequency but not amplitude of asynchronous, evoked mIPSCs in the presence of mGluR antagonists. (A) Representative traces showing stimulation-evoked, asychronous mIPSCs in control conditions (Sr^2+^-substituted bathing solutions for all experiments; see Materials and Methods) (C), in DHPG (D) and after 10 min. of washout (W). Cal. bars: y: 13 pA, x: 40 ms. (B) Numbers of asynchronous evoked mIPSCs (white bars; counted in 31 traces per cell within a 150-ms window beginning 200 ms after a field stimulus in *s. radiatum*). Gray bars: frequency of spontaneous mIPSCs measured before stimulation (background). Events were counted in control condition (C), during DHPG application (D) and DHPG washout (W). Numbers of evoked mIPSCs (n = 6 cells); control: 30.2±5.6, DHPG: 21.2±5.9, Wash: 22.2±5.6. Numbers of background mIPSCs (same cells): control: 16.3±3.4 DHPG: 13.6±3.2; Wash: 14.5±6.4. Asterisks: significant differences from control evoked responses, p<0.05. No other groups differed by ANOVA followed by multiple t-tests. (C) Cumulative frequency of the amplitude distribution of evoked mIPSCs from one cell – control  =  white circles, DHPG  =  black circles, wash  =  gray triangles. Results typical of 6 of 7 cells analyzed. Inset: Average of superimposed traces (n = 53) of evoked mIPSCs before (control) and during DHPG application - C: Control, D: DHPG. Cal. bars: y: 8.6 pA, x: 13 ms.

Since it does not affect excitatory transmission, Asc-08007-1-1 probably affects eIPSCs by a postsynaptic action on GABA_A_Rs or a presynaptic effect on GABA release. To distinguish between these possibilities, we switched to a strontium (Sr^2+^)-substituted [21]–[24] extracellular solution. Replacing extracellular Ca^2+^ with Sr^2+^ causes copious asynchronous quantal release of GABA (mIPSCs) after stimulation of inhibitory interneurons [24] (Fig. 6A). Quantal analysis of release can then be done by counting evoked mIPSCs and measuring their amplitudes. To control for the sporadic occurrence of spontaneous (not evoked) mIPSCs, which could conceivably confound the analysis, we also measured mIPSCs occurring in a 150-ms window prior to the stimulus. These ‘background’ mIPSCs (Fig. 6B) were not altered by Asc-08007-1-1, indicating that any measured changes in evoked mIPSCs were not contaminated by the background events. Application of the Asc-08007-1-1 for 5 min significantly decreased the frequency of evoked asynchronous mIPSCs (p<0.05, n = 7) during drug application; a 10-min washout was accompanied by partial recovery (p<0.05; Fig. 6B). Amplitude distributions were assessed via cumulative frequency plots followed by K-S tests. In contrast to the consistent reduction of mIPSC frequency in all cells, in 6 of 7 cells there was no significant reduction in the distribution of evoked asynchronous mIPSC amplitudes (e.g., Fig. 6C). We conclude that Asc-08007-1-1 reduces GABA release.

### A newer batch of Ascent Scientific DHPG did not elicit mGluR-independent effects

**Figure 7:**
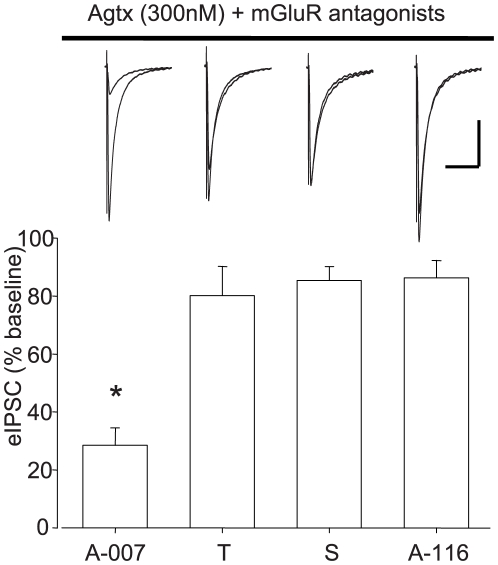
Asc-08116-5-3 DHPG does not cause mGluR-independent effects. Representative traces (top) and pooled data (bottom) showing the effects of 50 µM Asc-08116-5-3 DHPG for 5 min on eIPSCs in slices pretreated in ω-agatoxin IVA (300 nM) and YM298198 plus MPEP. Traces (black  =  baseline, dashed  =  DHPG) are representative averages of 10 consecutive responses in each condition. Peak eIPSC amplitude depressions expressed as percent baseline amplitudes: Asc-08007-1-1(A-007): 28.5±5.9%, n = 5; T-DHPG (T): 80.2±10.0%, n = 4; S-DHPG (S): 85.3±4.9%, n = 3; Asc-08116-5-3 (A-116): 86.2±6.1%, n = 6. Asterisk: significant difference from baseline responses, p<0.05. Cal. bars: y:200 pA, x: 100 ms.

To determine if the unusual actions of Ascent Scientific DHPG were unique properties of batch Asc-08007-1-1, or if they are common to all DHPG samples from this company, we tested batch Asc-08116-5-3 as well. In this series of experiments the slices had been pretreated in 300 nM ω-agatoxin IVA (agatoxin), to block GABA release from interneurons that release through P/Q type VGCC and do not express CB1Rs. As eCBs primarily inhibit release from interneurons that release GABA through N-type VGCC [25], agatoxin increases the relative contribution of GABA release from eCB-sensitive interneurons [26], [27], and thus provides a more sensitive assay for possible anomalous effects on these cells. We observed that, whereas group I mGluR antagonists YM298198 and MPEP fully blocked the effects of Asc-08116-5-3, Asc-08007-1-1 continued to reduce the eIPSCs to ∼28% of baseline values (p<0.01). The effects of T-DHPG and S-DHPG (Fig. 7) were also abolished by mGluR antagonists. This indicates that the factor responsible for mGluR-independent effects is not a universal property of (S)-3,5 DHPG from Ascent Scientific, but thus far has only been found in multiple samples from one batch.

### Chemical analysis of DHPG

**Figure 8:**
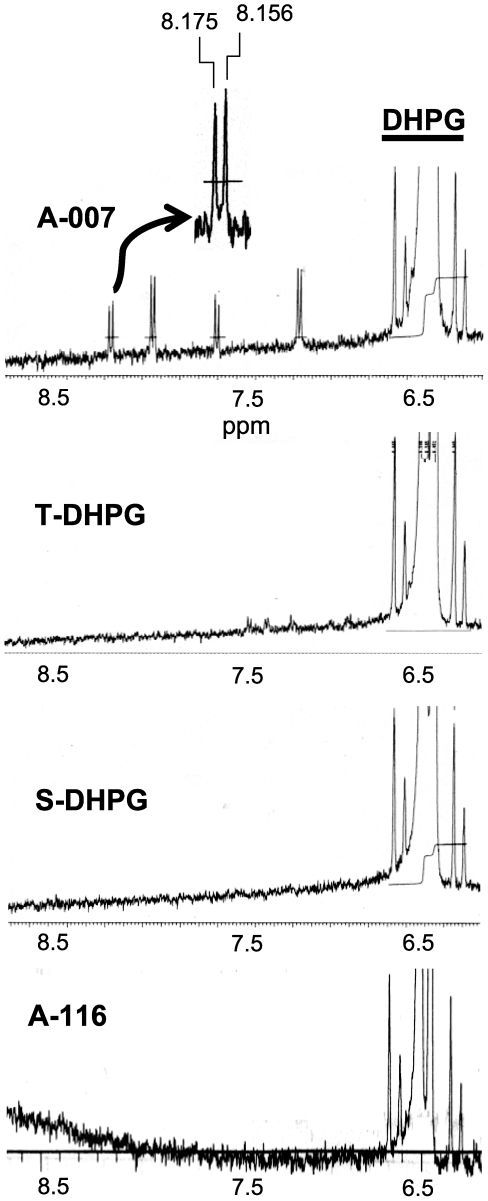
Distinctive peaks in proton NMR spectrum of Asc-08007-1-1 DHPG. Segments of 1D proton NMR spectra derived from samples of Asc-08007-1-1 (A-007), Tocris DHPG, Sigma DHPG, and Asc-08116-5-3 (A-116). The samples were prepared in D_2_O and the x-axis shows the chemical shifts in parts per million (ppm) with respect to the tetramethylsilane (TMS) reference signal at zero. The most striking difference between Asc-08007-1-1 and all other samples is the series of 4 doublets between about 7.2 and 8.2 ppm. The arrow in the top trace shows an enlargement of one of the doublets. Various organic molecules have chemical shifts in the range of 7.2 to 8.2 ppm, but the one responsible for the doublet pattern has not been identified. Not shown are the regions of the spectra between 0 and the water peak at 4.8 ppm in which irregular sequences of peaks were found in all samples. Each irregular sequence appeared to be unique for each preparation and did not obviously distinguish Asc-08007-1-1 from the others.

The data point to an mGluR- and CB1R-independent property of Asc-08007-1-1 that affects GABA release. One possibility is that there is a chemical factor in Asc-08007-1-1 that is responsible. Although a thorough chemical analysis of Asc-08007-1-1 was beyond the scope of this investigation, we did obtain both HPLC and proton NMR comparisons of T-DHPG, S-DHPG, Asc-08007-1-1, and Asc-08116-5-3. All analyses were carried out “blind”; i.e., the technicians had only a series of coded and otherwise unmarked samples. The code was broken by the authors after the analyses had been performed. The HPLC analysis was carried out by Ascent Scientific laboratory. By HPLC, samples of (S)-DHPG from all sources were essentially identical (data not shown). They were ≥98% pure; the racemate, (R)-DHPG constituted a small contaminant that was present in all samples, indicating that it could not account for the unusual effects. We also obtained proton-NMR spectra from the NMR facility at the University of Maryland School of Medicine. Two independent runs were done, and T-DHPG, S-DHPG, Asc-08116-5-3, and two different samples of Asc-08007-1-1, were tested. Unique, irregular minor peaks indicative of contaminants or impurities were present in all samples in the range from 0 to 4.8 ppm (data not shown). However, a uniform series of four doublets clearly distinguished the Asc-08007-1-1 sample from all other samples, including Asc-08116-5-3 (Fig. 8). Relative to the tetramethylsilane (TMS) reference signal at zero, the four doublets (perhaps a doublet of doublets) have chemical shifts of between 7.2 and 8.2 ppm. The doublet peaks appear to be correlated, suggesting they may be part of the same molecule. If they do represent one molecule, then estimating its relative abundance by integrating the areas under the peaks, and comparing the sum to the integrated peak areas associated with DHPG, suggests that this molecule could account for ∼1% of the sample. We do not know the identity of the molecule or indeed if it has any relationship to the anomalous properties of Asc-08007-1-1.

## Discussion

A batch of the widely used selective group I agonist, (S)-3,5 DHPG, Ascent Scientific Asc-08007-1-1, causes a major, reversible, suppression of GABAergic synaptic transmission that is evidently independent of mGluR and CB1R activation. In our hands, the action is not shared by preparations of the same drug from other companies, or by a different batch from Ascent Scientific. The observations are important because they reveal that a preparation of a widely used selective group I mGluR agonist that is ∼99% pure by HPLC and NMR can have pronounced non-specific effects. Moreover, the resemblance between our observations and published reports of anomalous mGluR agonist effects of DHPG suggests that our data could help reconcile the contradictory observations if the enigmatic action of DHPG were occasionally to be found in preparations from other sources. Among the controversial results are those pertaining to the mobilization of eCBs by mGluRs, an area of intense current research interest. Finally, in pointing to the existence of a factor that selectively reduces GABA release without affecting glutamate release, the data could lead to discovery of novel regulators of transmitter release.

DHPG is a selective group I mGluR agonist [28]. (S)-(+)-a-Amino-4-carboxy-2-methylbenzenacetic acid (LY367385) and MPEP are selective antagonists of mGluR1 and mGluR5, respectively, that in combination generally inhibit all effects of DHPG. However, antagonist-resistant DHPG actions have been reported. Activation of group I mGluRs can mobilize eCBs in hippocampus [8] and cerebellum [7]. However, some data show that while bath-applied DHPG rapidly suppresses eIPSCs, pretreatment of slices with a CB1R antagonist only reveals a reduction of eIPSC suppression after ≥10 min of DHPG treatment (e.g. [11] [19], Figs. 5C, 8B). DHPG must have reduced the eIPSCs by a CB1R-independent mechanism prior to this time. The results [11] would also suggest that the CB1R-independent suppression of IPSCs is caused by DHPG, but not by synaptically-released glutamate ([11], Figs. 4A, 5C). CB1R-independent effects like this are not always seen, however [7], [8], [12]. These discrepancies could be explained if an unrecognized mGluR- and CB1R-independent factor transiently suppressed eIPSCs and occluded the actual mGluR- and CB1R-dependent actions of DHPG. Unfortunately, it is not reported whether or not mGluR antagonists inhibited the DHPG-mediated responses [11], so this inference remains unverified.

Volk et al. [15] show that, although the combination of LY367385 and MPEP abolishes DHPG-induced LTD of fEPSPs as well as the phosphorylation of ERK, the antagonists leave untouched a large, transient, DHPG-induced fEPSP depression. The magnitude and time course of this antagonist-resistant effect are essentially identical to the anomalous IPSC suppression that we observe. Volk et al. [15] used either R,S- or S-DHPG obtained from Tocris, suggesting the possibility that effects like those seen with Asc-08007-1-1 are not unique, but may be associated with preparations from other sources. Note, however, that in our hands Asc-08007-1-1did not affect glutamatergic transmission.

We observe that the mGluR antagonist-resistant early eIPSC suppression caused by Asc-08007-1-1 is followed by the mGluR- and CB1R-antagonist-sensitive iLTD that is normally produced by DHPG or glutamate released by synaptic stimulation [10]. Similarly mGluR-dependent LTD is produced as usual after the early mGluR- or CB1R-independent phase in other work [11], [15]. In other words, even when DHPG samples do produce anomalous effects, they retain their expected efficacy at group I mGluRs. In our case, this is not surprising: Asc-08007-1-1 resembled all other compounds in HPLC and proton NMR analyses. Thus the anomalous effects must be attributed to an additional action, rather than an entirely different one. We do not know the origin of the additional activity. The HPLC results and the series of doublet peaks found uniquely in the proton NMR analysis of Asc-08007-1-1 are the only clues. If this contaminant is responsible, it must be quite potent in antagonizing GABA release, since by both chemical assays the purity of Asc-08007-1-1 is ∼99%. Given the broad use of DHPG in diverse areas of research, proper care must be taken to verify its specificity.

## Materials and Methods

### Animal Treatment and slice preparation

#### Ethics Statement

All animal handling work was conducted in accordance with national and international guidelines. All animal handling protocols were reviewed and approved by the University of Maryland School of Medicine IACUC. The number of animals used was minimized, and all necessary precautions were taken to mitigate pain or suffering. Five- to seven-week old Sprague–Dawley (Charles River) rats were deeply sedated with isoflurane and decapitated. Slices, 400-µm-thick, were cut on a Vibratome (model VT1200s Leica Microsystems) in an ice-cold bath solution and then stored at room temperature for 1 h before transfer to the recording chamber (RC-27L, Warner Instruments, CT, USA) at 30°C. The extracellular recording solution contained (in mM) 120 NaCl, 3 KCl, 2.5 CaCl_2_, 2 MgSO_4_, 1 NaH_2_PO_4_, 25 NaHCO_3_, and 20 glucose, and was bubbled with 95%O_2_, 5%CO_2_ (pH 7.4). Ionotropic glutamate responses were blocked with 2,3-Dioxo-6- nitro-1,2,3,4-tetrahydrobenzo[f]quinoxaline-7-sulfonamide (NBQX, 10 µM) and D-(−)-2-Amino-5-phosphonopentanoic acid (D-AP5, 20 µM). When they were used, the mGluR antagonists were bath-applied at the following concentrations: YM298198 – 4 µM, MPEP – 10 µM, LY341495 – 100 µM.

### Electrophysiology

Whole-cell pipettes were pulled from thin wall glass capillaries (1.5 O.D., World Precision Instruments, Florida, USA). They contained (in mM) either 146 KGluconate (KGluc), 1 NaCl, 1 MgSO_4_, 0.2 CaCl_2_, 2 EGTA, 10 HEPES, 4 MgATP, 0.3 tris GTP, or 90 CsCH_3_SO_4_, 1 MgCl_2_, 50 CsCl, 2 MgATP, 0.2 Cs_4_-BAPTA, 10 HEPES, 0.3 Tris GTP and 5 QX314. Electrode resistances in the bath were 3–6 MΩ. If the series resistance, when checked by a –5mV step, changed significantly (∼20%), the data were discarded. In recordings done with the KGluc-based electrode solution, pyramidal cells from the CA1 hippocampal *s. pyramidale* region were clamped at a holding potential of –50 mV. In experiments with the KCl-based electrode solution cells were held at −70 mV. Monosynaptic eIPSCs were elicited by 200-µs-long extracellular stimuli delivered at 0.25 Hz with concentric bipolar stimulating electrodes placed in *s. radiatum*. Data were collected using either an Axopatch 200B or an Axopatch 1C amplifier (Molecular Devices, Pennsylvania, USA), filtered at 2 kHz and digitized at 5 kHz using a Digidata 1322 (Molecular Devices) and Clampex 9.0 (Molecular Devices).

Asynchronous mIPSCs were recorded in the whole cell configuration described above with the KCl-based intracellular solution. After breaking into a cell, the extracellular solution was changed to one containing 4 mM MgSO_4_ and 4 mM strontium (Sr^2+^) instead of Ca^2+^. The amplitude and frequency of asynchronous sIPSCs that followed an evoked IPSC were measured 200 ms after the stimulation artifact, during a 150 ms windows for each trace (to obtain a sufficient number of events, data was gathered from 31 traces per condition), following the procedures of Morishita et al [24].

For experiments involving field excitatory postsynaptic potentials (fEPSPs), patch electrodes were filled with 2 M NaCl. The fEPSPs were recorded in *s. radiatum*, between CA3 and *subiculum*. Extracellular stimulation was given at 0.05 Hz with a bipolar electrode located in either *s. radiatum* near CA3 or *subiculum*. When fEPSPs were recorded in the presence of gabazine, the CA3 area was cut off to prevent the development of spontaneous epileptiform activity. In some cases, the extracellular solution was also changed to one containing, in mM: 120 NaCl, 2.4 KCl, 6 MgSO_4_, 1 NaH_2_PO_4_, 25 NaHCO_3_, 20 glucose, 1.5 CaCl_2_, as well as AP5 10 µM and NBQX 0.01 µM, which helped suppress hyperexcitability. When tested without gabazine, this extracellular solution did not to affect our basic results.

### Chemicals

(S)-3,5 DHPG was obtained from Tocris Bioscience (Missouri, USA) batch 26, Sigma-Aldrich (Missouri, USA), batch 087k46202, and Ascent Scientific (Bristol, UK), batches Asc- 08007-1-1 and Asc -08116-5-3. All drugs were made up as 1000X stocks in distilled water (except for AM251 and SR141716, which were made at a 10000X concentration in DMSO) as soon as they were obtained. Stocks were immediately divided into 20 µL aliquots and frozen at −20°C until use. Once thawed, aliquots were either used or discarded; none were refrozen and reused. Care was taken to see that all samples were handled identically, and were used within two months after preparation. AM251 and stocks were dissolved in DMSO. Final concentration of DMSO in the bath was 0.01%. Drugs were obtained from Tocris Bioscience (AM251, MPEP, SR95531, and LY341495), Ascent Scientific (NBQX, AP5, and YM298198), and NIDA (SR141716). All other drugs and chemicals were purchased from Sigma-Aldrich.

### Data Analysis

Statistical tests among groups were done with one-way ANOVA with repeated measures (two way ANOVA for the data presented in Fig. 1) followed by a Student- Newman-Keuls (SNK) test (SigmaStat). Paired t tests were used for single comparisons. The significance level for all tests was p<0.05 (*), except the Komolgorov-Smirnov (K-S) tests in Fig. 7 where the significance level was 0.005. Group mean±SEMs are shown for display purposes. Measurements of mIPSC frequency and amplitude (Fig. 6) were performed with the Mini Analysis Program (Synaptosoft, New Jersey, USA). For comparison of cumulative amplitude distributions (mIPSCs experiments) we used the Kolmogorov-Smirnov test, available at http://www.physics.csbsju.edu/stats/KS-test.n.plot_form.html. In Figures 1–3 the group data for experimental time courses were smoothed by running averages (n = 10), and incremental sampling (n^th^  = 10) in SigmaPlot 11.0.
